# Reversal of third-degree AV block with ruxolitinib in VEXAS-associated myocarditis: a case report

**DOI:** 10.1093/ehjcr/ytaf580

**Published:** 2025-11-11

**Authors:** Saif Younas, Jan Van Beveren, Jan Bogaert, Mariëlle Beckers, Joris Ector

**Affiliations:** Department of Cardiology, University Hospitals Leuven, Herestraat 49, Leuven 3000, Belgium; Department of Cardiology, University Hospitals Leuven, Herestraat 49, Leuven 3000, Belgium; Department of Radiology, University Hospitals Leuven, Herestraat 49, Leuven 3000, Belgium; Department of Hematology, University Hospitals Leuven, Herestraat 49, Leuven 3000, Belgium; Department of Cardiology, University Hospitals Leuven, Herestraat 49, Leuven 3000, Belgium

**Keywords:** VEXAS syndrome, Myocarditis, Atrioventricular block, Bradycardia, Ruxolitinib, Autoinflammatory disease, Case report

## Abstract

**Background:**

VEXAS syndrome is a newly described autoinflammatory condition; cardiac involvement is exceedingly rare. This case illustrates a reversible complete heart block due to myocarditis in VEXAS, successfully treated with targeted therapy.

**Case summary:**

A 69-year-old man with known VEXAS syndrome presented with dizziness and syncope. Workup revealed third-degree atrioventricular (AV) block and imaging consistent with myocarditis. Standard therapy (atropine, isoprenaline infusion) stabilized the patient, and ruxolitinib (a JAK inhibitor) was initiated alongside corticosteroids to treat the underlying inflammatory syndrome. Within days, the patient’s AV conduction was restored without the need for pacemaker insertion. He was discharged with improved cardiac function and ongoing immunomodulatory therapy.

**Discussion:**

Targeted immunomodulation can reverse cardiac conduction block caused by inflammation in VEXAS syndrome. This case underscores the importance of recognizing autoinflammatory syndromes as a cause of heart block and suggests a potential role for JAK inhibitors in treating inflammatory myocarditis.

Learning points

- Myocarditis due to VEXAS syndrome can present with high-degree atrioventricular (AV) block, a rare but reversible cardiac complication.
- Targeted immunomodulatory therapy with Ruxolitinib may restore AV conduction and prevent permanent pacemaker implantation.
- Multidisciplinary assessment and advanced imaging, such as cardiac MRI, are critical for diagnosing and managing inflammatory cardiac involvement in autoinflammatory syndromes.

## Introduction

VEXAS (vacuoles, E1 enzyme, X-linked, autoinflammatory, somatic) syndrome, caused by somatic UBA1 mutations in haematopoietic stem cells, affects mainly adult males and presents with systemic inflammation and haematologic abnormalities. Common features include fever, chondritis, pulmonary disease, vasculitis, neutrophilic dermatitis, and macrocytic anaemia.^1,2^ Treatments include glucocorticoids, anti-IL-6 agents, JAK inhibitors, and azacytidine; stem cell transplantation is the only curative option in selected patients.^1^ Cardiovascular involvement is rare, but cases of pericarditis, myocarditis, and vasculopathy are reported.^3,4^ This case illustrates the rare occurrence of third-degree atrioventricular (AV) block caused by myocarditis in VEXAS syndrome and its resolution after initiation of ruxolitinib.

## Summary figure

**Figure:**
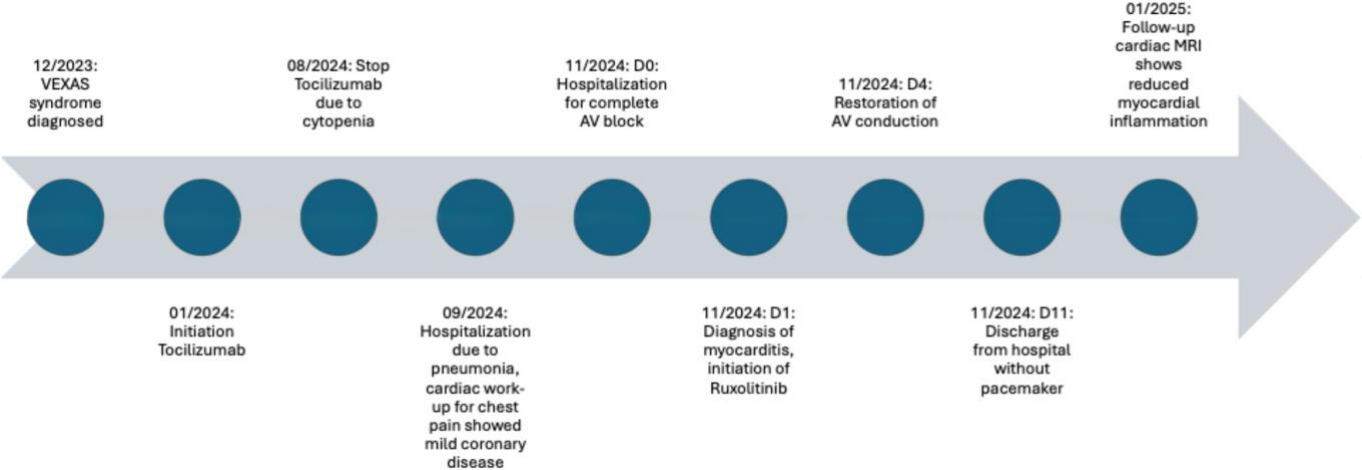


## Case presentation

### Patient information

**Figure 1:**
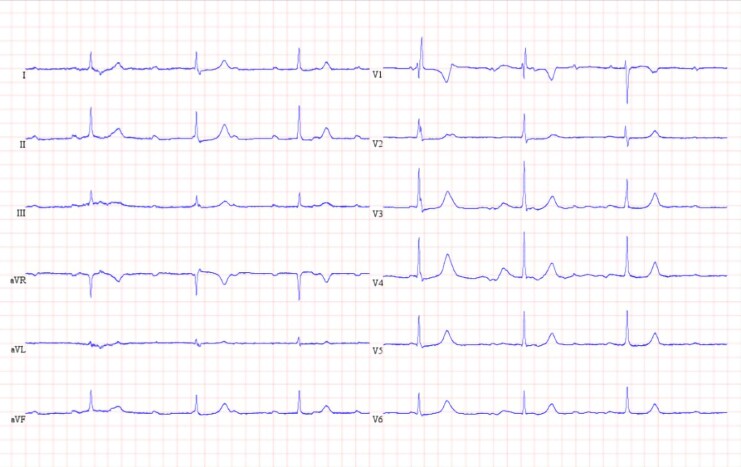
ECG at time of presentation showed third-degree AV block with a junctional escape rhythm at 40 bpm with intermittent RBBB morphology as seen evidently in lead V1. (ECG, electrocardiogram; AV, atrioventricular; RBBB, right bundle branch block).

**Figure 2:**
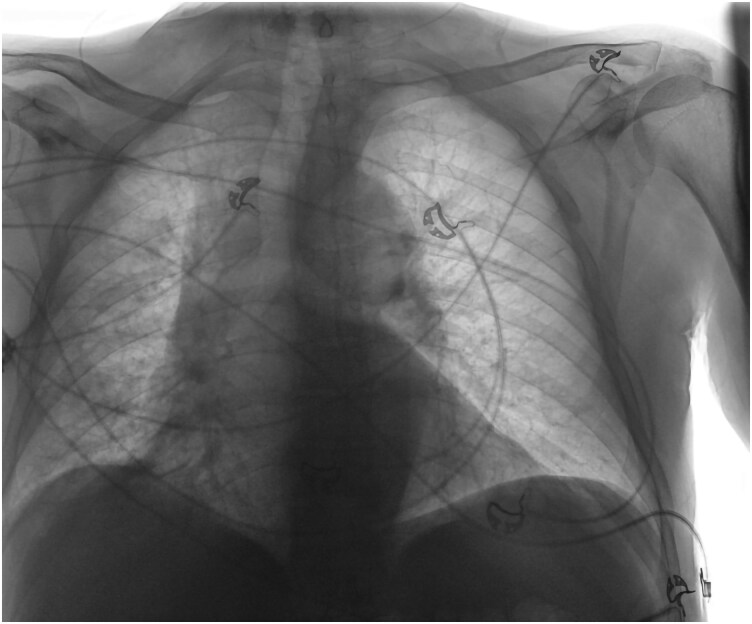
Chest radiograph at time of presentation demonstrated findings suggestive of pulmonary congestion, with an area suspicious for an underlying infiltrative process in the right middle lobe.

**Figure 3:**
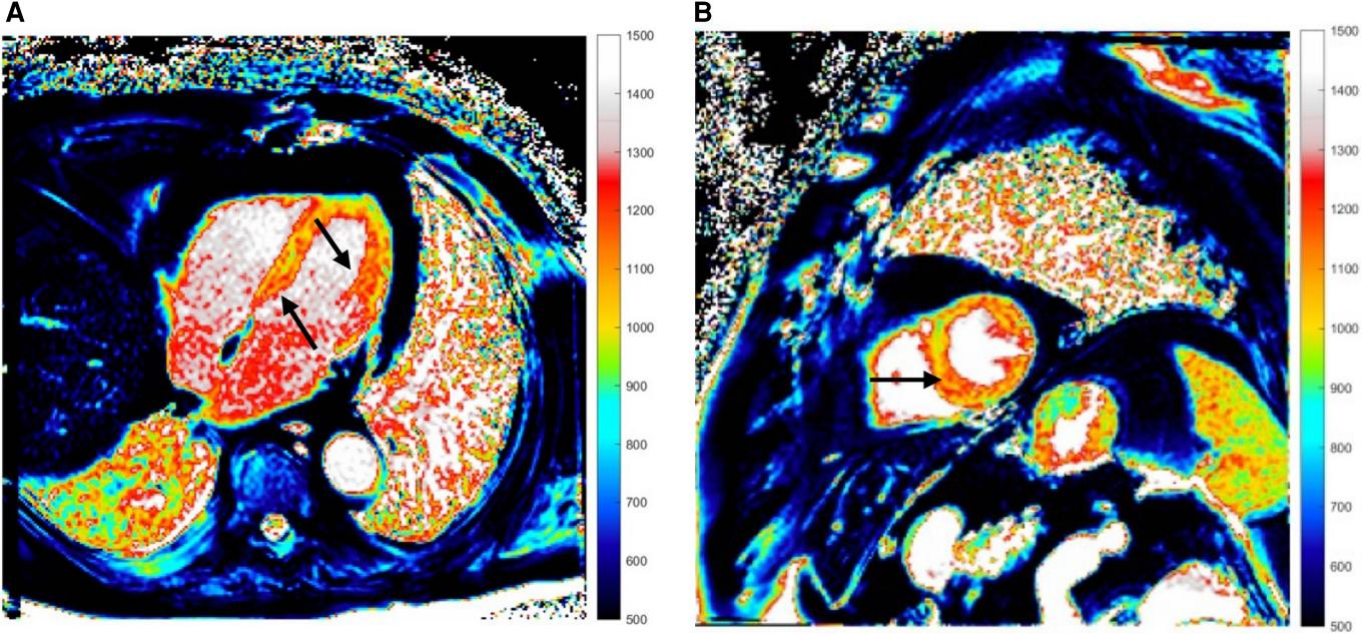
Cardiac MRI native T1 long and midventricular short axis view during the initial hospitalization. Normal myocardial native T1 at 1.5T is ∼965–1025 ms. Diffusely increased values, highlighted by black arrows, indicated myocardial oedema and inflammation (MRI, magnetic resonance imaging; ms, milliseconds).

**Figure 4:**
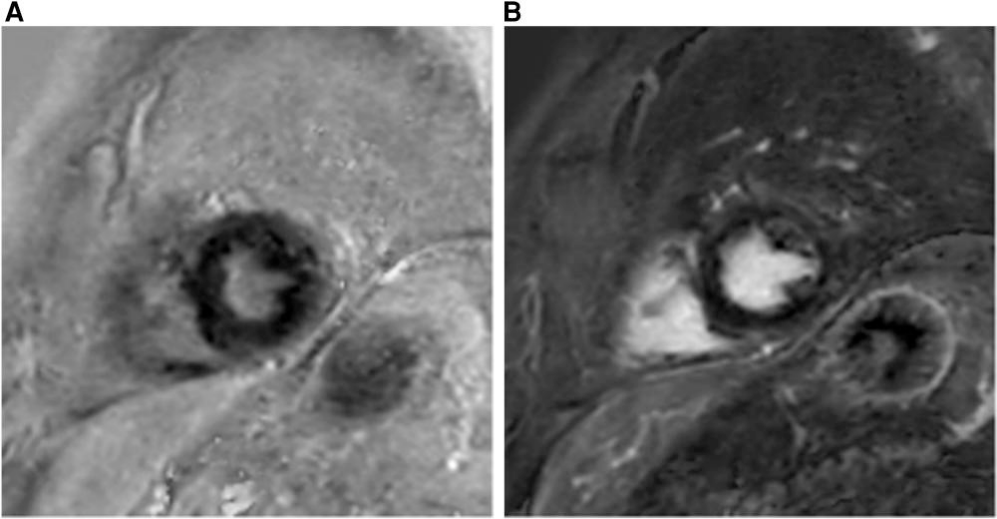
Baseline cardiac MRI in midventricular short axis view during initial hospitalization and at follow-up without evidence of LGE (MRI, magnetic resonance imaging; LGE, late gadolinium enhancement).

A 69-year-old male with a history of dyslipidaemia was diagnosed with VEXAS syndrome in December 2023 via genetic testing. His family and social history were unremarkable. He presented with recurrent fevers, skin and pulmonary lesions, polychondritis, and deep venous thrombosis at the time of diagnosis. He was treated with methylprednisolone intermittently from August 2022 and continuously after diagnosis in December 2023. Tocilizumab was added in January 2024. Inflammation was resolved under this combination, but tocilizumab was stopped in August 2024 due to cytopenia. A month after his last tocilizumab dose, he was hospitalized for pneumonia. During admission, chest pain with elevated troponin (105 ng/L) and anteroseptal T-wave inversion prompted cardiac evaluation, revealing normal ultrasound and moderate ostial RCA disease on angiography. In November 2024, he presented to the emergency department with dizziness, near-syncope, dyspnoea, and general weakness. On examination, his blood pressure was 100/41 mmHg with a pulse of 42 b.p.m. He showed signs of heart failure with peripheral oedema, elevated jugular venous pressure, and a respiratory rate of 22 b.p.m. with an oxygen saturation of 88%. Electrocardiogram showed third-degree AV block with a junctional escape rhythm at 40 b.p.m. (*Figure 1*). Laboratory findings showed mild troponin elevation at 52 ng/L, macrocytic anaemia, and elevated inflammatory parameters with CRP at 151.6 mg/L and NT-proBNP at 22 889 ng/L. No major electrolyte disturbances or drug-related causes of bradycardia were identified. Chest radiograph suggested pulmonary congestion with a possible right middle lobe infiltrate (*Figure 2*). Chest computed tomography showed marked improvement in right lung ground-glass opacities and irregular consolidations compared to the recent hospitalization. Echocardiography showed preserved left ventricular function, dilated right ventricle with severe tricuspid regurgitation, moderate pulmonary hypertension, and moderate mitral regurgitation with elevated filling pressures, consistent with bradycardia-related acute heart failure. No valvular vegetations were seen on transoesophageal echocardiography. Coronary angiography was done during a prior admission and showed only moderate CAD, so no acute ischaemic cause for the AV block was identified. Cardiac magnetic resonance imaging (MRI) revealed a heterogeneous increase in native T1/T2 and extracellular volume values within the myocardium, with elevated native T1 of 1078–1166 ms (site normal 965–1025 ms) and elevated T2 of 60 ms (normal 56 ms) (*Figure 3*). Notably, there was no late gadolinium enhancement (LGE) to suggest myocardial fibrosis or necrosis (*Figure 4*). According to the 2018 Lake Louise Criteria, the presence of both T1-based (non-ischaemic injury) and T2-based (oedema) abnormalities was consistent with active, non-ischaemic myocardial inflammation.

**Figure 5:**
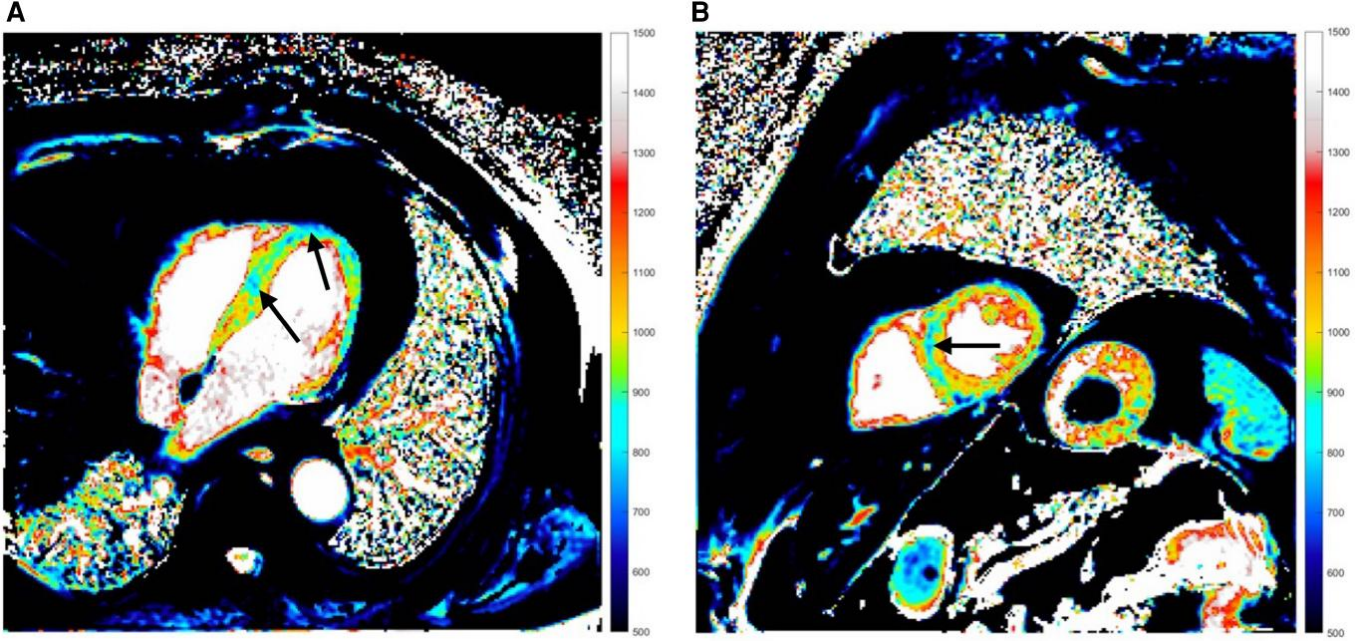
Follow-up cardiac MRI native T1 long and midventricular short axis views. Compared with normal T1 values, unusually low T1 signals were observed in certain areas, highlighted by black arrows, suggestive of iron deposition in the myocardium and liver (MRI, magnetic resonance imaging).

Due to the absence of coronary occlusion and signs of infarction, ischaemic conduction system disease was eliminated as a cause for the AV block. Degenerative AV nodal disease was also deemed unlikely given the acute presentation and patient’s systemic illness. Infiltrative diseases like sarcoidosis or amyloidosis were considered, but the patient’s known VEXAS and MRI lacking sarcoid-pattern LGE made these less probable, with amyloidosis seeming less likely due to the acute onset. Infectious myocarditis (Lyme) was considered, but Borrelia serology was negative. An endomyocardial biopsy (EMB) was contemplated, but given the MRI results and increased procedural risk, it was deemed unnecessary. The patient’s inflammatory syndrome provided a plausible explanation. Considering the aforementioned points, myocarditis related to VEXAS syndrome was supported as the cause of complete AV block in this patient. The patient was initially treated with atropine. Following this, an isoprenaline infusion was administered, which effectively increased the heart rate. Given an immediate chronotropic response to isoprenaline, we managed the bradyarrhythmia with continuous monitoring and vasoactive support. A temporary transvenous pacemaker was not placed because haemodynamics remained adequate after chronotropic support. Due to the initial suspicion of an infectious process, empirical antibiotic therapy was initiated and continued for 5 days. Acute heart failure was managed with loop diuretics. The dose of methylprednisolone was maintained at 16 mg daily, consistent with the regimen used over the preceding 5 months. Ruxolitinib (a JAK inhibitor) was introduced on the following day at a dose of 15 mg twice daily orally, following recommendations from the haematology team. The decision to initiate ruxolitinib was made as a steroid-sparing measure, aimed at compensating for the discontinuation of tocilizumab due to the patient’s intolerance to this medication. Over the subsequent days, the patient’s heart rhythm returned to sinus, and the isoprenaline infusion was gradually tapered and discontinued by the fifth day post-admission. The decision not to implant a permanent pacemaker diverged from standard practice, based on the anticipated improvement with ruxolitinib. At discharge, the patient exhibited resolution of AV block and significant clinical improvement. Follow-up imaging 3 months post-admission revealed a clear decrease in native T1 and T2 values, indicating the resolution of myocardial oedema and inflammation. This provided objective evidence of improved myocarditis. However, there was a persistently elevated ECV and unusually low T1 signals in certain areas, suggesting iron deposition in the myocardium and liver (*Figure 5*). This could be attributed to chronic inflammation or transfusions leading to iron overload in the context of VEXAS syndrome. Further evaluation via iron quantification on MRI was advised. The patient remains stable on immunomodulatory therapy.

## Discussion

This case underscores the potential of immunomodulatory therapy in addressing high-degree AV block caused by inflammation in VEXAS syndrome. The prompt identification that the heart block was likely inflammatory, given the patient’s history, enabled targeted therapy rather than immediate permanent pacing. The link between AV block and myocarditis has been previously documented.^5^ Utilizing advanced imaging techniques, such as cardiac MRI, to confirm myocarditis and guide therapy proved advantageous. The multi-disciplinary approach involving rheumatology, haematology, and cardiology collaboration contributed to the positive outcome. This case also enhances the literature by documenting a new organ involvement in VEXAS syndrome and its management. Although the temporal relationship between ruxolitinib initiation and restoration of sinus rhythm strongly suggests a therapeutic effect, causality cannot be definitively established in the absence of a rechallenge or mechanistic study. It remains possible that spontaneous resolution or the concurrent corticosteroid therapy contributed to the reversal of the AV block. A limitation of this case is the absence of an EMB, which remains the gold standard for definitive diagnosis of myocarditis. However, we did not pursue EMB for the following reasons: (i) the patient had active systemic inflammation and cytopenia, increasing procedural risk; (ii) cardiac MRI provided strong non-invasive evidence of acute inflammatory myocarditis; (iii) there was rapid clinical and biomarker improvement with anti-inflammatory therapy; and (iv) the result was unlikely to change acute management beyond immunosuppression. While this approach is justifiable in clinical practice, it limits histopathological validation of the underlying myocardial process. Iron-overload cardiomyopathy, a recognized cause of both conduction disease and heart failure, could therefore not be definitively excluded. Nonetheless, the CMR findings and the patient’s rapid clinical improvement under immunosuppressive therapy strongly suggest that VEXAS-related inflammation was the predominant underlying mechanism. Moreover, the rarity of VEXAS syndrome implies limited availability of established diagnostic and management protocols in this setting.

To our knowledge, this is the first report suggesting a possible role for ruxolitinib in the treatment of cardiac involvement in VEXAS. Although ruxolitinib is commonly used in the treatment of haematologic disorders, its effectiveness has been described previously in cases of steroid-refractory checkpoint-inhibitor myocarditis.^6^ In summary, this case suggests that disease-directed immunomodulation may allow recovery from high-grade AV block when inflammation is the main mechanism. Given the absence of histology and potential confounders, we view ruxolitinib as a plausible therapeutic modality in VEXAS-associated myocarditis. Further investigation is needed to assess for true treatment effect.

## Lead author biography


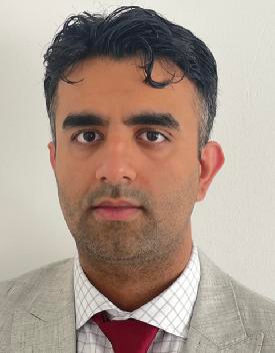


Dr. Saif Younas is a final-year cardiology resident at University Hospitals Leuven, Belgium. He has a particular interest in heart failure, cardiac intensive care, and interventional cardiology.

## Data Availability

The data underlying this article are available within the article itself. No additional datasets were generated or analysed.
